# Extracellular Matrix Component Remodeling in Respiratory Diseases: What Has Been Found in Clinical and Experimental Studies?

**DOI:** 10.3390/cells8040342

**Published:** 2019-04-11

**Authors:** Juliana T. Ito, Juliana D. Lourenço, Renato F. Righetti, Iolanda F.L.C. Tibério, Carla M. Prado, Fernanda D.T.Q.S. Lopes

**Affiliations:** 1 Department of Clinical Medicine, Laboratory of Experimental Therapeutics/LIM-20, School of Medicine of University of Sao Paulo, Sao Paulo 01246-903, Brazil; jutiyakito@hotmail.com (J.T.I.); juliana.dl31@gmail.com (J.D.L.); refragar@gmail.com (R.F.R.); iocalvo@uol.com.br (I.F.L.C.T.); 2 Rehabilitation service, Sírio-Libanês Hospital, Sao Paulo 01308-050, Brazil; 3 Department of Bioscience, Laboratory of Studies in Pulmonary Inflammation, Federal University of Sao Paulo, Santos 11015-020, Brazil; cmaximoprado@gmail.com

**Keywords:** extracellular matrix, lung function, asthma, chronic obstructive pulmonary disease, acute respiratory distress syndrome

## Abstract

Changes in extracellular matrix (ECM) components in the lungs are associated with the progression of respiratory diseases, such as asthma, chronic obstructive pulmonary disease (COPD), and acute respiratory distress syndrome (ARDS). Experimental and clinical studies have revealed that structural changes in ECM components occur under chronic inflammatory conditions, and these changes are associated with impaired lung function. In bronchial asthma, elastic and collagen fiber remodeling, mostly in the airway walls, is associated with an increase in mucus secretion, leading to airway hyperreactivity. In COPD, changes in collagen subtypes I and III and elastin, interfere with the mechanical properties of the lungs, and are believed to play a pivotal role in decreased lung elasticity, during emphysema progression. In ARDS, interstitial edema is often accompanied by excessive deposition of fibronectin and collagen subtypes I and III, which can lead to respiratory failure in the intensive care unit. This review uses experimental models and human studies to describe how inflammatory conditions and ECM remodeling contribute to the loss of lung function in these respiratory diseases.

## 1. The Connective Tissue of the Lung 

Extracellular matrix (ECM) components are constituents of the connective tissue that play essential structural roles in maintaining organ functionality. The composition of the connective tissue is determined by a hierarchical molecular organization; under inflammatory conditions, this organization depends on the balance between the injury and remodeling of these components, which can lead to chemical and structural changes and reduced tissue functionality [1].

In lung tissues, the main components of the ECM are elastic and collagen fibers, proteoglycans, glycoproteins, and metalloproteinases (MMPs), and their tissue inhibitors (TIMPs). Among these components, collagen is the most abundant [1,2,3,4], and the number of collagen fibers is, thus, the primary determinant for the mechanical properties of the lungs [1].

There are more than twenty different subtypes of collagen molecules, and in the lung parenchyma, subtypes I and III mostly constitute the structural framework of the alveolar walls, whereas subtype IV is present in the basement membranes [5]. The fibers of collagen subtype I are stiffer than those of collagen subtype III (as indicated by a comparative histology [6]), and the subtype I/III ratio determines the resistance of these fibers to breakdown under mechanical forces during stretching [1]. 

Elastic fibers are considered the main component responsible for the elastic recoil properties of the lungs, and these fibers are composed of at least two morphologically distinguishable components—elastin and microfibrils [7,8]. Elastic fibers are mechanically connected to collagen [9] by microfibrils or proteoglycans [10,11], with elastin acting predominantly in pulmonary elasticity, in the presence of normal breathing lung volumes [12], while collagen acts progressively in the presence of volumes that approach the total lung capacity [13].

Microfibrils are composed of fibrillins, microfibril-associated glycoproteins, and transforming growth factor-beta (TGF-β) binding proteins, among which fibrillins are the major components, providing a scaffold for elastin polymer aggregation [7]. 

Proteoglycans are composed of a protein core and side chains of glycosaminoglycans [2], which are a family of linear polysaccharides that constitute the ECM and basement membrane [14,15]. Glycosaminoglycans play a pivotal role in the maintenance of the collagen fiber assembly, as well as water balance, cell adhesion and migration [5].

The major cells responsible for the ECM production and normal ECM turnover are fibroblasts [16,17,18,19]. Different molecules can influence the activity of these cells, such as cytokines, growth factors, and components of the ECM itself. Among the relevant cytokines, TGF-β plays a pivotal role in inducing the production of the ECM components by fibroblasts [16].

Additionally, ECM components usually interact with epithelial cells, serving as ligands to transmit signals to regulate adhesion, migration, proliferation, apoptosis, survival, or differentiation. In addition, they can release growth factors and other signaling molecules that regulate cells behavior [20].

**Figure 1:**
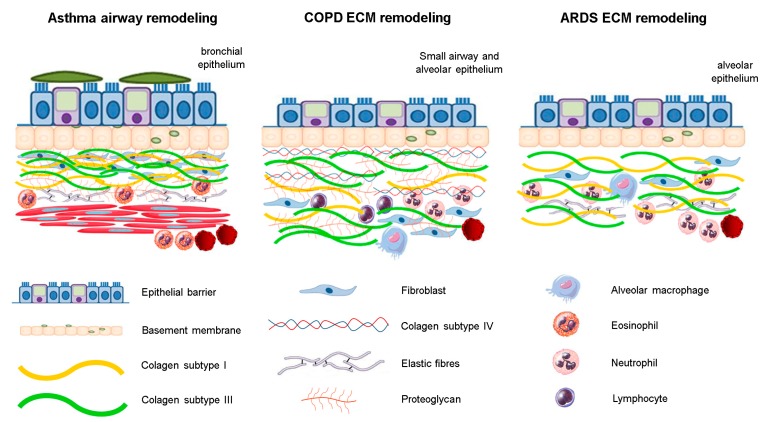
The structural changes of ECM components in respiratory diseases. There is an inflammatory process associated with different fibers rearrangement. In asthma, structural changes are mainly in the bronchial epithelium, whereas in ARDS, these are observed near the alveolar epithelium. In COPD, the ECM remodeling is observed predominantly in small airways and distal areas of parenchyma.

Under pathological conditions, ECM turnover is altered [18,21,22], leading to an altered architecture and consequent failure of the mechanical properties of this tissue (Figure 1) [16]. Considering the importance of the altered composition of the ECM components, under chronic inflammatory conditions for lung function impairment due to respiratory diseases, in this review, we summarized how structural changes in some ECM components can impact the worsening of lung function in three lung diseases—asthma, chronic obstructive pulmonary disease (COPD), and acute respiratory distress syndrome (ARDS).

## 2. Airway Remodeling in Asthma

A recent study confirmed that asthma rates are increasing in countries outside of the United States; approximately 300 million people or 4.3% of the world’s population suffer from asthma [23]. The prevalence of asthma increased 2.9% each year from 2001 to 2010 (20.3 million people in the United States had asthma in 2001, compared with 25.7 million in 2010) [24]. The Global Initiative for Asthma estimates that there will be an additional 100 million people with asthma, by the year 2025 [25].

Asthma is a common and potentially severe chronic disease that can be controlled but not cured. It presents symptoms such as, wheezing, shortness of breath, tightness in the chest, and coughing that vary over time, in terms of their occurrence, frequency, and intensity. Asthma is associated with variable expiratory airflow, due to bronchoconstriction, thickening of the airway wall, and mucus. Symptoms can be triggered or worsened by factors such as viral infections, allergens, smoking, exercise, and stress [25]. 

Inhaled allergens come into contact with the respiratory mucosa and are captured by dendritic cells present in the bronchial epithelium. These cells recognize and process the antigen and present it to T helper (Th) lymphocytes [26]. These cells release mediators and recruit other inflammatory cells into the lung. After these events, the transformation of CD4+ T cells into different profiles occurs. Interleukins (IL)-4, IL-5, and IL-13, induce the proliferation of Th2 cells, interferon-gamma (IFN-γ), and IL-2 induce the proliferation of Th1 cells, and transforming growth factor-beta (TGF-β), and IL-6 induce the proliferation of Th17 cells [27,28].

The inflammatory process in asthma, results in chronic inflammation of the airway walls and lung tissue, eventually triggering bronchoconstriction and structural changes, called airway remodeling [29]. However, several lines of evidence have shown the role of mechanical forces that occur during bronchoconstriction in inflammation-independent airway remodeling [30,31,32,33,34]. Grainge et al. suggest that repeated bronchoconstriction in asthma induces epithelial stress and initiates a tissue response that leads to structural airway changes [35]. 

In addition, a few studies have addressed the initial occurrence of airway wall remodeling in young to very young children. Pohunek et al. showed evidence for ECM remodeling very early in childhood. In a bronchial biopsy study of 27 children, aged 1.2 to 11.7 years, with chronic respiratory symptoms, the thickness of the subepithelial lamina reticularis was observed to be greater in children with bronchial asthma diagnosed at follow-up, compared with children who did not progress to asthma. This suggests that remodeling might be present even before asthma becomes symptomatic [36]. Corroborating this study, a biopsy study in pre-school children with severe wheezing, reported a number of characteristics of airway remodeling, such as increased basement membrane thickness, increase in airway smooth muscle (ASM), vascularity, and mucus gland area, without any relationship with the inflammatory cell count [37]. These continuous tissue adaptations trigger changes in lung structure, geometry, and tissue properties [38], and they are considered the main causes of the symptoms associated with a decreased lung function [39]. Hill et al. showed in a theoretical model that the inflammatory conditions lead to mechanical stresses, leading to the release of contractile agonists, exacerbating the fiber remodeling in ASM [20]. This process explains why some asthmatic patients present partial and irreversible loss of respiratory function, over time, especially severe asthmatic patients who experience an accelerated decline in pulmonary function, with the disease progression [40]. The changes that occur during airway remodeling consists of subepithelial reticular basement membrane (RBM) thickening, increased ASM thickness, angiogenesis, and goblet cell hyperplasia associated with irreversible loss of lung function [41]. Airway remodeling and subepithelial fibrosis are not inhibited in severe asthma, despite corticosteroid treatment and might lead to worsening of symptoms as the disease progresses [42,43].

The hyperresponsiveness of airways leads to exaggerated airway narrowing which is result of the ASM contraction. Thus, it is important to understand the changes that occur in the ECM that surround the airways, as well as the interactions between the ECM and the ASM [44,45]. Khan et al. demonstrated a significant loss of tethering (interaction of ASM to its surrounding parenchyma) forces in mouse lung microsections incubated overnight with proteolytic enzymes, through two different in vitro experiments [46,47]. In the first study, the authors exposed slices from control animals to porcine pancreatic elastase (PPE) and evaluated the cholinergic responses, in order to verify the hyperresponsiveness, by treating with acetylcholine. The elastase exposure resulted in increased magnitudes and velocities of airway narrowing, impaired relaxation, and increased rupture of the airways, from the surrounding parenchyma [46]. In the second study, the authors treated mouse lung slices with PPE, collagenase, or both, and assessed the hyperresponsiveness, by adding acetylcholine. The treatment with PPE or collagenase increased the lumen narrowing induced by acetylcholine even more. When treated with both proteases together, there was an increase in the velocity of contraction, as well as a decrease in the velocity of relaxation, resulting in a retraction of the airway and a reduction in the tethering forces [47].

### 2.1. Clinical Studies

In asthmatic patients, smooth muscle mass is increased, due to a coordinated increase in hyperplasia, hypertrophy, and ASM cells. Smooth muscle cells actively participate in inflammatory and remodeling processes, through the release of pro-inflammatory cytokines, chemokines, and ECM proteins; therefore, these cells might contribute to the pathogenesis of asthma [48]. 

The migration of smooth muscle cells is a recently described feature of airway remodeling. Joubert et al. showed that chemokines have the ability to induce human ASM cell migration and to increase their contractility, revealing another process that could significantly contribute to the overall airflow obstruction in these patients [49]. James et al. showed that human lung smooth muscle in the airways of patients with asthma, must shorten by only 40% of its resting length, in order to completely occlude the airway lumen. These results are consistent with observations made during bronchial challenge [50]. While in normal subjects, the decrease in forced expiratory volume in one second (FEV_1_) reaches a plateau, in patients with asthma, the FEV_1_ continues to decrease without reaching a plateau [51]. Smooth muscle mass has been correlated with asthma severity [52].

Yick et al. evaluated the spirometry and methacholine responsiveness associated with positive staining of elastin, collagen I, III, and IV, decorin, versican, fibronectin, laminin, and tenascin, in ASM performed in atopic mild asthma and healthy subjects. In this study, ECM in ASM was related to the dynamics of airway function in the absence of differences in ECM expression between asthma and the controls. This indicates that the ASM layer in its full composition, is a major structural component, in determining variable airways obstruction in asthma [45]. In addition, Slats et al. showed that hyperresponsiveness is associated with the level of expression of α-smooth muscle-actin, desmin, and elastin, within the bronchial wall, but not with myosin, calponin, vimentin, type III collagen, or fibronectin. This suggests that expression of each of the contractile and structural smooth muscle proteins, as well as components of the ECM, distinctly influences the dynamic airway function [44].

Airway mucus contains approximately 2% mucins, but some exogenous factors (antigen contact) and endogenous mediators, produced by inflammatory and structural cells, can contribute to submucosal and goblet cell hyperplasia, airway mucus hypersecretion, and upregulation of the MUC protein expression, to varying degrees. However, submucosal gland hyperplasia and goblet cell hyperplasia are observed in the airways of asthmatic patients and are a feature that is particularly evident in fatal asthma [53].

Asthmatic lung biopsies have shown that airway fibroblasts are morphologically distinct from lung parenchymal fibroblasts. Distal lung tissue fibroblasts are broader with more cytoplasmic projections, and airway fibroblasts synthesize more procollagen type I, after TGF-β stimulation [54]. These structural differences might explain, at least partially, why a variety of repair responses are observed in the proximal and distal airways, in response to a damaging stimulus in the lung parenchyma of asthmatic patients.

Produced in response to the cytokine TGF-β, collagen is a potential marker of remodeling in asthma [55]. It has been documented that in moderate (FEV_1_ 60–80% predicted) and severe asthma (FEV_1_ ≤ 60% predicted), there is an increase in the deposition of fibroblasts, collagen I, and collagen III, in bronchial biopsies [42,52]. In addition to its involvement in the inflammatory process, TGF-β is also involved in the changes observed in the ECM, stimulating the production of fibroblasts, collagens type I and III, fibronectin, and proteoglycans [56].

Lung ECM remodeling in asthma is determined by the rate of ongoing deposition and degradation of proteins, including collagens I, III, and V, fibronectin, tenascin, lumican, and biglycan. These components are likely secreted due to the activation of fibroblasts and myofibroblasts, mainly because of the TGF-β signaling [57,58]. Collagen fibers are the most abundant elements of the ECM in the lung, constituting approximately 70% of the lung tissue, and changing the structure, quantity, or geometry of their distribution can trigger changes in lung functioning [58,59,60].

Considered important in the remodeling process, proteoglycans (decorin, biglycan, and lumican) play roles in the interaction of fibrils and collagen fibrogenesis in the tissue, with other components of the ECM [61]. The increase in decorin expression in patients with severe asthma, could be a protective mechanism for modulating pulmonary remodeling. Conversely, an increased amount of decorin could regulate and stabilize collagen spacing and create a more rigid collagen matrix, which might affect the overall elasticity of the lung tissue [62].

Proteoglycans are formed by the linkage of glycosaminoglycans, such as heparin and heparan sulphate, which play important roles in allergic and inflammatory processes, such as asthma. This molecule binds to chemokines, interleukins, growth factor, and other proteins [14,63], and promotes the recruitment of leukocytes, playing a critical role in airway hyperresponsiveness and inflammation [64]. In asthma, heparin can regulate bronchial hyperresponsiveness, influence the inflammatory process, by inhibiting the recruitment of inflammatory cells, and attenuate tissue damage by binding and neutralizing chemokines and cytokines that are released from inflammatory cells [14,65].

In fatal asthma, which is characterized as clinically severe asthma, fibronectin levels, and elastic fibers are increased in the smooth muscle of the airway wall [66]. Other abnormalities in airway matrix structure in asthma, include a specific increase in the lumican and biglycan isoforms, which is also associated with tissue injury and worsening of the lung function (FEV_1_ ≤ 80% predicted) [67]. MMPs play an important role during the remodeling process, by degrading the components of the ECM (especially MMP-9 and MMP-12). The inhibition of the activated MMPs might be rapidly conducted by TIMPs that are produced by most mesenchymal cells [68]. Righetti et al. and Pigati et al. showed that in a model of chronic allergic inflammation, increases in MMP-9 and TIMP-1 were associated with increases in the volume fraction of actin, collagen, and elastic fibers in the airway and the distal parenchyma, revealing the importance of the parenchyma, during the remodeling process [60,69].

Although the remodeling process is potentially harmful to lung function, this response is thought to be an attempt to protect against aggressive pulmonary inflammation. Previous evidence has shown that without tissue remodeling, the patient can experience worsening of symptoms and a faster decline in lung function [70].

As the main inhaled medication used in asthma, several studies have reported the effects of glucocorticoids on airway remodeling, including the reduction of RBM thickness [71,72], improvements in the number of ciliated epithelial cells [73], and decreases in both vessel numbers and percent vascularity in the submucosa [72,74]. However, it is important to note that although some patients under corticosteroids treatment show improvement in symptoms and inflammatory cell numbers, within the sputum of the airways, the improvement in airway hyperresponsiveness to nonspecific stimuli, occurs only after much longer periods of treatment [71,75]. Elliot et al. showed in post-mortem airway sections from asthmatic patients that, some structural changes that lead the hyperresponsiveness might be partly independent of inflammation and, therefore, are not reversible by anti-inflammatory treatment [76].

Alternatively, bronchial thermoplasty is a novel non-drug therapy that targets airway remodeling [77]. Evidence has shown that this technique reduces the ASM area and that this response is correlated with improvements in asthma control and quality of life, and decreases severe exacerbations and hospitalizations [78,79].

### 2.2. Experimental Models

Experimental allergic asthma in mice is a reliable and clinically relevant model of human disease, because clinical studies of asthma are not able to clarify all aspects of disease pathophysiology [80]. Several experimental protocols and animal species have been used in experimental models of asthma, such as cats, dogs, rats, guinea pigs, pigs, equines, and primates [81,82]. However, the most common species studied in the recent decades have been guinea pigs and mice (particularly BALB/c mice) [83]. The experimental protocols for inducing asthma include two phases—(i) sensitization is achieved by the intraperitoneal or subcutaneous route in mice and by inhalation in guinea pigs (associated with intranasal instillation of antigen), which has been increasingly used because human asthma is induced by inhalation of antigen; and (ii) antigen challenges are performed through intratracheal and intranasal instillation and aerosol inhalation [84]. The classic antigens used in experimental models of asthma are ovalbumin (OVA) and house dust mites [57,58,81]. 

Several studies using these experimental models of asthma have shown functional alterations in the resistance and elasticity of the respiratory system associated with inflammatory eosinophilic infiltrates; expression of Th2 and Th17 cytokines, MMP-9-, MMP-12-, TIMP-1-, and TGF-β-positive cells; increased deposition of actin, collagen, and elastic fibers, and increased mucus production in the airways and lung tissues [57,59,60,69,85].

Airway resistance is resistance to the in- and outflow of air, exerted by the airway walls and lung elasticity. Also known as elastic resistance, the reciprocal of lung compliance is the pressure change required to elicit a unit of volume change; both parameters represent lung function [86]. Hyperresponsiveness is a feature of asthmatic rats that indicates functional changes in airway resistance and lung elasticity [87]. Measures of airway resistance and lung elasticity are commonly used in experiments, to evaluate hyperresponsiveness in respiratory disease models, including asthma models [85,88,89].

In a guinea pig asthma model sensitized with OVA, Possa et al. showed positive correlations between the volume fractions of actin, collagen, and elastic fibers, and airway resistance and elastance [48]. A decrease in the actin deposition in the airways, reduces the resistance and elastance of the respiratory system, as a result of antigen sensitization. Corroborating this study, Vasconcelos et al. demonstrated in guinea pigs with chronic allergic inflammation that the concomitant reduction of airway hyperresponsiveness and smooth muscle mass are correlated, suggesting that such structural changes could explain the functional change in smooth muscle contractile responsiveness [90]. Additionally, Righetti et al., in the same experimental model, showed that increased actin, collagen, and elastic fibers in the lung tissue are associated with functional alterations in the alveolar lung tissue mechanics [60]. Therefore, airway remodeling in experimental models resembles the pathophysiological features of human asthma [76].

Other components of the ECM appear to participate in airway remodeling in human and experimental models. In a mouse model of asthma exacerbated with lipopolysaccharide (LPS), Camargo et al. showed an increased number of cells positive for MMP-9, MMP-12, TIMP-1, and TGF-β, as well as an increased volume fraction of collagen fibers I and III, decorin, actin, biglycan, lumican, and fibronectin in the lung tissue [57]. These animals were also treated with an anti-IL-17 antibody and showed a decreased pulmonary inflammation, edema, and airway remodeling, compared to the non-treated animals [57]. In a murine asthma model, Dos Santos et al. showed that the presence of the IL-17 and Rho-kinase (ROCK) proteins, enhances the percentage of maximal increase in the respiratory system resistance and elastance, after being challenged with methacholine. Additionally, there were increases in the number of cells positive for ROCK1, ROCK2, TGF-β, MMP-9, MMP-12, and TIMP-1, and the percentages of isoprostane, biglycan, decorin, fibronectin, and collagen fibers, in the asthma group. However, all these changes were attenuated after treatment with an anti-IL-17 antibody or a ROCK inhibitor, and the combination of these treatments potentiated this protective effect [58]. 

Some of the mediators mentioned above also elicit early mucus hypersecretion [91]. Pardo–Saganta et al. showed increases in the expression of mucous cell-specific genes and the number of ciliated cells in the murine pseudostratified airway epithelium, after the OVA challenge [92]. Asthmatic patients showed higher levels of MUC5AC in the airways and more total mucus, with consequences for pulmonary function [93,94]. 

These associations in humans were supported in the OVA-sensitized guinea pigs, which exhibited increases in lung tissue resistance and elastance, eosinophilic infiltration in the airways and parenchyma, a significant increase in collagen density, and a concurrent parenchymal contractile response [95]. Almeida–Reis et al. showed in an experimental model of asthma that chronic allergic lung inflammation reduces mucociliary clearance, due to alterations in the rheological properties of mucus, increasing acidity, wettability, and adhesiveness of the mucus [85]. The functional consequences of these abnormalities, mostly result in increased airway wall thickness, sputum production, and airway narrowing, due to sputum secretion [25].

Experimental models have been developed to better understand these mechanisms, to evaluate both the safety and efficacy of therapies, before clinical trials, and to mimic the pathophysiology of human disease [96]. Table 1 summarizes lung function results and markers of airway remodeling from clinical and experimental studies of asthma.

## 3. Extracellular Matrix Remodeling in COPD

COPD is a common, preventable, and treatable disease, characterized by persistent respiratory symptoms and airflow limitations, caused by a mixture of small airway disease (e.g., obstructive bronchiolitis) and parenchymal destruction (emphysema), mainly induced by smoke exposure [97]. These changes do not always occur together, and there are some variations in the degree of airway disease and emphysema in COPD patients, which might explain the heterogeneity of the response to treatment [98]. Although long-acting bronchodilators have been used in the management of COPD, these drugs are not efficient to control the inflammatory process, as well as the structural changes [99].

Persistence of the inflammatory process leads to structural changes, such as parenchymal tissue destruction (resulting in emphysema) and disruption of normal repair and defense mechanisms (resulting in small airway fibrosis), culminating in a decrease in lung elastic recoil, gas trapping, and progressive airflow limitation [97].

The level of obstruction in COPD patients is determined by spirometry, in which a post-bronchodilator value of FEV_1_/forced vital capacity (FVC) < 0.70 confirms the presence of persistent airflow limitation. Table 2 shows the severity of lung function impairment in COPD patients, based on post-bronchodilator FEV_1_ [97].

The chronic inflammatory response in COPD patients is associated with increased numbers of inflammatory cells, such as macrophages, neutrophils, and CD4+ and CD8+ T lymphocytes [100], and fibroblasts in the airways, which play a pivotal role in the upregulation of proteases, such as MMPs, resulting in the destruction and remodeling of ECM components, in the small airways [101] and in the parenchyma [102]. The role of collagenases, such as MMP-1, MMP-8, and MMP-13, in ECM fiber destruction in COPD patients, has been described [103,104], and MMP-12 has been the most commonly described collagenase in experimental models [105,106,107]. In response to fiber destruction by MMPs, there is a structural reorganization of parenchymal fibers, constituting a dynamic process of repair and remodeling [108]. It is believed that changes in major lung ECM components, such as collagen subtypes I and III, and elastin, are involved in the loss of elasticity, during emphysema progression [1,109,110,111].

Although emphysema is defined by the destruction of distal air spaces, with or without fibrosis [97], the majority of clinical studies have described an increase in the amount of ECM fiber deposition in the airways and the lung parenchyma [112,113,114,115]. However, it is important to emphasize that these evaluations were usually performed in patients who were in advanced stages of the disease and in experimental models, after a few days of disease induction [104,116,117,118].

### 3.1. Clinical Studies

In COPD patients, the majority of studies have shown that structural changes in the airway walls are associated with disease progression. The ECM composition and the amount of different constituents are altered in these patients [22,119,120]. Kranenburg et al. showed that these changes occurred mainly in the surface epithelial basement membrane and were characterized by increased deposition of collagen subtypes I, III, and IV, associated with high levels of collagen subtypes I and III, in both the bronchial lamina propria and adventitia, as well as enhanced expression of fibronectin in the vascular intima [22]. Additionally, in this study, a significant, direct correlation was demonstrated between the severity of COPD (moderate and severe stages) and an enhanced expression of these different ECM components. 

Several observations about COPD patients have pointed out the importance of examining the airway smooth muscle and its interaction with the surrounding parenchyma (tethering), since the loss of elastic tissue observed in the ECM of COPD patients [121,122] can reduce tethering forces around the airway, resulting in a higher propensity airway narrowing [123,124]. Chen et al. demonstrated in vitro that ASM cells from COPD patients stimulated with cigarette smoke (CS) extract, have higher deposition of collagen type VIII alpha I, but no differences on the deposition of collagen V and fibronectin [125].

Hogg et al. [126] demonstrated that thickening of the airway walls, by the remodeling process, was strongly associated with the progression of obstruction. In addition, the authors showed that the accumulation of inflammatory cells (polymorpho-nuclear leukocytes, macrophages, CD4+ and CD8+ T lymphocytes and B cells) in the lumen of the airways, leads to a malfunction of the mucociliary clearance apparatus [108,126].

It is worth noting that there is no consensus on the number of ECM components in the airways and parenchyma in COPD patients. In moderate COPD patients, Annoni et al. showed a decrease in elastic fibers, collagen subtype I, and versican, in small and large airways, associated with a higher fibronectin fractional area [5]. Additionally, these authors suggested that a decrease in elastic fibers, leads to a loss of airway parenchyma, resulting in airway collapse and gas trapping [5]. Such findings are consistent with those of previous studies, in which the authors showed a decrease in elastic fibers, in both the small airways and the alveolar septa, in lung samples from COPD patients, who were in moderate stages, and showed severe lung function impairment [122,127].

In contrast, in lung samples from surgically resected lobes, Vlahovic et al. previously demonstrated an increase in the volume of the alveolar septum, with a parallel increase in elastic fibers in the COPD patients, with mild to moderate emphysema. Additionally, they observed increased numbers of interstitial fibroblasts and macrophages [112].

Although there has been divergence among studies regarding the different ECM components, there is a consensus about the structural changes in these components, which usually involve fragmentation [5,122]. Abraham and Hogg showed that severe disruption and remodeling of the elastic and collagen fibers, in alveolar airspaces of emphysematous human lung samples and collagen, spreads to alveolar airspaces, indicating extensive alterations in the collagen fiber structures, in the alveolar region [108]. 

In vitro studies have addressed some of the potential mechanisms that drive the ECM component remodeling. In this context, Sun et al. showed an increased immunoreactivity of LL-37, a protein of the human cathelicidin family, which is involved in the tissue remodeling processes, in the small airway epithelium of COPD patients, compared to healthy smokers. They showed that the expression of LL-37 in the airway epithelium, was correlated with the airway wall thickness, as well as a deposition of collagen in the airway walls. Additionally, the authors showed in vitro, that exposure to CS, induced an increase in LL-37 and augmented the fibroblast collagen production [128]. 

Milara et al. showed that CS exposure induces chronic lung remodeling in differentiated bronchial epithelial cells, from smokers and moderate COPD patients. These cells undergo mesenchymal transition, as a result of the release of TGF-β1, by enhancing oxidative stress, the phosphorylation of ERK1/2, and SMAD3, and the downregulation of cyclic monophosphate (cAMP) [129]. A similar response was found in all airway wall compartments of smokers and patients with COPD, but mostly in actively-smoking COPD subjects [130].

Anti-TGF-β treatments can attenuate CS-induced lung injury in COPD. Both, in vitro and in vivo studies indicate that, inhibition of TGF-β signaling can protect the lungs from altered lung morphology, impaired lung function, and lung injury [131,132]. 

Brandsma et al. demonstrated differential effects of fluticasone treatment on different lung compartments, in severe COPD patients [98]. This inhaled steroid stimulated the production of decorin by airway fibroblasts, inducing the restoration of decorin around the airways; while in contrast, in parenchymal fibroblasts, fluticasone inhibited the production of biglycan and procollagen, indicating inhibition of tissue repair in emphysematous areas. The authors attribute this response to the phenotypic differences between lung fibroblasts, including ECM production and the response to TGF-β [19,98]. Since not all treatments are able to reverse tissue damage in COPD, some ECM protein markers have been used in determining disease prognosis. Some findings have demonstrated that serological markers can reflect the extent of structural changes in COPD patients. Sand et al. showed that serological biomarkers of collagen subtypes I, III, IV, and VI were associated with an increased mortality [133]. The increased serum levels of procollagen type I, associated with high levels of IL-6 and IL-8, in COPD patients, might indicate the airway remodeling condition, as the inflammatory process plays an important role in stimulating collagen synthesis [134]. Vignola et al. demonstrated that increased levels of active elastase and overproduction of TIMP-1, relative to MMP-9, were associated with the magnitude of lung changes on high-resolution computed tomography [135]. Papakonstantinou et al. showed that hyaluronic acid levels in the serum of COPD patients are associated with COPD severity and airflow limitation, pointing out this molecule as a potent target to control airway inflammation and remodeling in COPD [136].

### 3.2. Experimental Models 

Since the majority of studies in COPD patients have been restricted to lung samples obtained from pulmonary biopsy or resection, experimental models have been used to understand how abnormal fiber repair, under COPD conditions, could interfere with lung functionality [137].

There are many ways to induce COPD in rodents. The administration of proteases, such as PPE, and exposure to CS, remain the most commonly used strategies to induce lung structural changes that resemble those observed in COPD patients [138]. 

The CS-induced model is considered to best represent human COPD, since CS is the main risk factor for this disease in humans [139,140]. Several studies have demonstrated parenchymal destruction and remodeling, worsening lung function, and inflammatory processes, over a long period of time, after CS exposure [140,141,142,143]. In addition to these characteristic features of COPD, Beckett et al. showed systemic effects on the skeletal muscle and the heart, in a short-term model of COPD [144].

Conversely, the elastase-induced model requires a short time to induce drastic structural changes, compared to the CS-induced model; for this reason, it is the most commonly used model to study how changes in the ECM fiber deposition in the parenchyma, interfere with respiratory mechanics [141,145].

It is important to note that both models show an inflammatory process characterized mainly by increased macrophages [143,146], but neutrophils and the presence of CD4+ and CD8+ T lymphocytes have also been observed. MMP-12 is the metalloprotease most often described in animal models of COPD, and along with TGF-β, it acts by modulating increases in the amounts of elastic and collagen fibers [143,147]. Structural changes are observed mainly in the lung parenchyma, where increased alveolar enlargement is observed, reflecting alveolar wall destruction, and the presence of fragmented elastic and collagen fibers [145]. 

The use of proteinase inhibitors represents a potential therapeutic treatment for emphysema in animal models. Lourenço et al. showed that treatment with a serine protease inhibitor from *Rhipicephalus (B.) microplus* (rBmTI-A), decreased the MMP-12+ cells, and the collagen fibers in the lung parenchyma, and reversed the loss of elastic recoil and alveolar enlargement, in an emphysema model induced by PPE instillation and CS exposure [143,146]. Similarly, the use of other proteinase inhibitors, also reduced the elastase-induced pulmonary inflammation, remodeling, oxidative stress, and mechanical alterations [148,149].

There are differences in the ECM fiber remodeling patterns between experimental models. Although all newly deposited fibers showed a fragmented appearance, there was an increase in collagen subtype III, with no differences in the collagen subtype I, in the lung parenchyma, and there were differences between the experimental models, when we analyzed the elastic fiber components. In addition, in the CS model, we observed increased fibrillin amounts, while in the PPE-induced model, there was an increase in elastin [145].

Robertoni et al. [150] showed that a reduction in ECM fibers preceded increased deposition of these fibers in the distal parenchyma in a PPE-induced model, suggesting that in this animal model, the destruction and repair processes did not occur simultaneously. These authors showed an initial decrease in the volume proportions of collagen subtype I (on the 3rd day) and subtype III (from the sixth hour until the third day), after the detection of increased MMP-8 and MMP-13 gene expression. On the twenty-first day, there was an increase in the volume proportion of collagen subtype III, and collagen subtype I returned to levels similar to those in the control groups. Additionally, MMP-12 gene expression was increased (from the third hour to the sixth hour) before the decrease in the volume proportion of elastin on the third day, with a subsequent increase in the proportion of this fiber on the twenty-first day. An increase in polymorphonuclear cells was observed beginning, in the first hour, after the PPE instillation, which remained until the third day [150].

The majority of studies describing the impact of ECM fiber remodeling on lung function impairment have emphasized the importance of collagen fibers [1,151,152]. Previous findings have demonstrated that fiber stiffness depends on the relative amounts of subtype I and subtype III collagens, since subtype I collagen is stiffer than subtype III [1,6]. Such findings might explain why, in animal models of emphysema, collagen fibers break at tensions that correspond to those recorded with normal breathing [152]. The stimulation of ECM components, such as collagen subtype I and subtype III, induced the proliferation, migration, and adhesion of ASM cells in rat models of COPD. The concomitant increase in TGF-β expression in these cells, induces overproduction of multiple ECM proteins, which might result in ASM cell hyperplasia [153].

The newly synthetized collagen fibers had altered configurations and were likely to be weaker, compromising the strength of the alveolar walls [152]. To determine how these structural changes impact the lung function, Kononov et al. used an elastase-induced model, to show that, the mechanical forces generated during normal breathing were sufficient to promote tissue damage and stress failure in the remodeled alveolar walls, with increased collagen and elastin [110]. Additionally, Ito et al. showed that parenchymal fibers failed at lower stress, after remodeling in mice, due to four weeks of PPE instillation, and the authors attributed these changes to the newly synthesized collagen fibers [152].

Recently, structural changes have been detected, prior to functional changes. In a PPE-induced model, although structural changes were detected in earlier stages of emphysema development (6 h after PPE instillation), significant decreases in tissue elastance and tissue resistance were observed only twenty-one days after elastase instillation [146,150]. In the CS-induced model, exposure required three months to induce a decrease in tissue elastance and tissue resistance, whereas alveolar enlargement could be detected after one month, with 30 min of exposure, repeated two times per day, for 5 days per week [142,154].

It is interesting that lung function parameters do not reflect the structural changes in COPD animal models, mainly due to technical difficulties in performing evaluations in small animals [86,155]. To detect changes in lung function in animal models, it is necessary to show significant changes in lung structure, which explains why many studies have not shown modifications in lung function but have conducted morphometric analysis [115,155,156]. Therefore, it is important to understand how these structural and functional changes occur at different time points, during disease development in experimental models, to facilitate the choice of the best model, according to the approach and goals. Table 3 summarizes lung function changes and markers of ECM remodeling in clinical and experimental studies of COPD.

## 4. Extracellular Matrix Remodeling in ARDS

ARDS was defined in 1994, but in 2011, after an initiative of the European Respiratory Society of Intensive Care Medicine endorsed by the American Thoracic Society, this disease was redefined by the Berlin definition. Three categories of ARDS were proposed, based on the degree of hypoxemia—mild (200 mm Hg < PaO_2_/FIO_2_ ≤ 300 mm Hg); moderate (100 mm Hg < PaO_2_/FIO_2_ ≤ 200 mm Hg); and severe (PaO_2_/FIO_2_ ≤ 100 mmHg).

ARDS remains an important cause of death within intensive care units, and approximately 30% of patients die due to ARDS, despite advances in therapeutic strategies [157,158]. Pulmonary fibroproliferation has been associated with higher mortality and ventilator dependence, and it remains an observable clinical feature, in a subset of patients [159].

In this context, there is increasing interest in better understanding the basic and pathophysiological mechanisms that drive the fibroproliferative response in ARDS. The initial site of the lesion is the alveolar epithelium or the endothelium [160,161].

The acute phase of ARDS is characterized by local and systemic inflammatory responses [162], and involves the release of several pro-inflammatory cytokines, such as tumor necrosis factor-alpha (TNF-α), IL-1β, and IL-8 [162,163,164,165]. Additionally, neutrophil infiltration, interstitial edema and hypoxemia, are often accompanied by aggressive ECM remodeling [166]. Chen et al. used in vitro models of acid-induced lung epithelial cell injury, to show that the interaction of these cells with monocytes, accelerates the epithelial remodeling process through EMT signaling [167].

Although the physiopathological mechanisms of ECM remodeling among asthma, COPD and ARDS are completely different, the ECM remodeling also requires the action of mechanical forces generated by the migration or contraction of myofibroblasts, by themselves, and the presence of fibronectin, initially produced by macrophages, which is responsible for the adhesion of cells to the matrix. At the end of the acute phase of ARDS, fibronectin is already being produced by myofibroblasts [168].

The fibroproliferative phase, which is mainly characterized by thickening of the alveolar wall associated with interstitial edema and large cellularity, occurs between 7 and 15 days after the primary injury. The cells most involved in this phase are neutrophils, macrophages, myofibroblasts, and type II pneumocytes [169]. The hyaline membrane that arises during the acute phase plays an important role in the fibroproliferative phase of ARDS, since it attaches fibronectin produced by alveolar macrophages to its surface.

Myofibroblasts deposit elements of the collagen and elastic systems, both, in the lumen and inside the alveolar septum, as well as in the walls of the blood vessels. Initially, there is an increase in the deposition of thin fibers of subtype III collagen. Many patients present resolution of the process at this stage, but some progress to the phase of fibrotic remodeling [170,171,172].

In later stages of this disease, thickening of the vessel wall is present, making gas exchange and local metabolism even more difficult. The main characteristic of this phase is a change in the gene expression of subtype I collagen, which is synthesized in increasing amounts. At the same time, there is an increase in collagenase-digested subtype III collagen (secreted in the previous phases), leading to a tendency towards the accumulation of fibrous tissue, in later stages of ARDS.

Tissue repair includes a variety of mechanisms, as well as edema reabsorption, resolution of inflammation, and cell proliferation, with the aim of repairing the alveolar epithelium [173].

During the fibrotic remodeling phase of ARDS (late phase), there is a trend towards increased deposition of elastic fibers in the alveolar septa, leading to a progressive fibroelastosis [172]. During this stage, alveoli obliterated by fibrosis are adjacent to the ectatic alveoli, with irregular, thickened walls covered by stratified epithelium, or simple columnar epithelium, likely derived from the bronchioles. In the alveolar spaces, there is a large number of pulmonary surfactants produced by numerous type II pneumocytes, which remain active after differentiating from type I pneumocytes, promoting alveolar re-epithelization.

The increase in the number of elastic fibers in the late stages of ARDS might be a compensatory response to the fragmentation and degradation of pre-existing fibers, in the early stages of the process. However, the deposition of large numbers of elastic fibers, leads to progressive elastosis, which is partially responsible for the loss of the normal architecture of the alveolar wall, contributing to the tendency to collapse [172,174].

Since the deposition of microfibrils precedes the appearance of elastin, it should be considered that, during the “de novo” synthesis process of elastic fibers, there will be a stage during which the areas undergoing remodeling will be rich in bundles of microfibrils, with very little or no elastin. Thus, in addition to the absence of the elastic component, the mechanical properties of these inextensible microfibrils, add to those of collagen I, yielding even more tissue resistance to the physical adaptations necessary for a good respiratory performance.

These structural alterations in the ECM have repercussions for the compliance of the pulmonary parenchyma, with impacts on the respiratory mechanics. Evidence has suggested that intrinsic factors, such as genetic patterns of inflammatory response modulation, can influence the production of interleukins involved in the disease progression [175]. Additionally, external factors, such as alcoholism, have been identified as promoters of inflammation and fibrogenesis in ARDS [176,177,178].

### 4.1. Clinical Studies

Pioneering studies performed by different groups in the 1990s have showed that, 72 h after the diagnosis of ARDS, patients already showed important increases in collagen synthesis, as detected by high levels of the N-terminal peptide of type III procollagen [179,180,181]. Additionally, these elevated levels are associated with histological lung fibroproliferation and mortality, in ARDS patients [169].

Although the alveolar level of N-terminal peptide of type III procollagen is considered the best surrogate marker for the diagnosis of lung fibroproliferation, Hamon et al. demonstrated that patients with active lung proliferation have higher fibrosis score, as evaluated by a chest CT scan, which allows an alternative use of this radiological tool, which is less invasive than fibroscopic bronchoalveolar lavage [182]. Thille et al. have showed that histological features of the lungs are related to the duration of ARDS. The authors analyzed 159 patients and found a reduction in the prevalence of exudative changes over time, with greater changes in patients with ARDS, for less than one week, and smaller changes in patients with a disease duration, between one and three weeks. However, the prevalence of proliferative changes increased over time and was greater in patients with a long duration of disease. These authors have also showed that fibrosis was more common in patients whose ARDS origin was pulmonary [183].

Interestingly, an almost complete recovery of lung function has been demonstrated in survivors of ARDS, after 6 to 12 months of evolution [184,185]. However, approximately 30% to 40% of ARDS patients in the late phase, evolve to an exacerbated and progressive remodeling process, culminating in the destruction of the pulmonary architecture and death [172]. In ARDS survivors, a negative correlation has been described between fibroproliferation and quality of life. Burnham et al. studied 82 patients with ARDS and showed that reduced lung compliance measured at the bedside, was associated with radiologic fibroproliferation, 14 days post-diagnosis [166]. These data were interesting since they could be helpful in identifying patients with ARDS who are at risk for complications in clinical conditions.

Zheng et al. showed the protective effects of ResolvinD1, a lipid mediator which attenuates the excessive polymorphonuclear infiltration and transmigration, in the fibroproliferative phase of ARDS. The authors demonstrated that ResolvinD1 inhibited primary human lung fibroblast proliferation, collagen production, and myofibroblast differentiation induced by TGF-β, from ARDS patients [186].

Although lung remodeling is an important feature in ARDS patients and is related to the deterioration of lung function, the assessment of lung repair in patients, remains limited. However, such assessments could be of great interest because of the prognostic relevance of lung repair in ARDS patients.

Unfortunately, no pharmacological agents that focus specifically on fibroproliferation are available at this time for the treatment of ARDS [166]. In this context, basic and experimental studies are relevant and could contribute not only to a better understanding of the fibroproliferative process in ARDS, but also to the development of new therapeutic strategies for ARDS patients.

### 4.2. Experimental Models

Most experimental models used to study ARDS investigated the acute phase, although at this time, most authors, including our group, have shown deposition of collagen fibers in the alveolar septa.

Once the direct or indirect etiological stimulus has ceased, the behavior of tissue remodeling is completely different. In animals subjected to direct lesions, we observed continuous deposition of collagen that remained stable, until the eighth week, followed by a deposition of elastic fibers, with significant differences after the first week. In animals subjected to an indirect initial insult, the levels of collagen deposition fell to basal levels, during the first week, after the insult, and elastosis was not observed [174].

We used animals challenged with LPS, and evaluated them 6 and 24 h after injury. We found that at 6 h, there was intense inflammation in the lung with high levels of pro-inflammatory cytokines; however, no signs of lung remodeling and no deterioration of lung function were detected at this time. Only at 24 h after LPS instillation did we observe an intense deposition of collagen fibers in the alveolar septa, and a reduction in the respiratory system and lung tissue compliance [187].

Costa et al. developed an experimental model of ARDS, induced by nebulized LPS, and they found that, 24 h after LPS, the animals showed increased pro-inflammatory cytokine levels, increased total septal volume, and a thickening associated with reduced surface density of the alveolar septa. However, after five weeks, the animals showed an increased total lung volume and accentuated collagen deposition, particularly collagen subtype I, associated with reduced MMP-2 protein expression [188]. This model could contribute to a better understanding of the remodeling process in ARDS, and the development of preventive or therapeutic strategies, to counteract lung remodeling in ARDS.

LPS is a widely used model to mimic ARDS alterations in experimental animals. In this regard, Oliveira et al. using in vitro techniques, showed that, LPS increased lung epithelial cell stiffness and is associated to cytoskeletal remodeling [189].

Although no studies in patients have investigated the effects of drugs in lung remodeling, in animals, we showed the effects of different pathways involved in remodeling. An extensive body of literature shows that natural substances can reduce acute lung injury (ALI) in animal models; however, few studies have focused on lung remodeling. In this regard, Mernak et al. showed that sakuranetin, a flavonoid that can significantly reduce lung inflammation, reduced collagen deposition in the parenchyma, when it was administered 6 h after LPS, the point at which animals have intense inflammation. Moreover, this compound also improved the lung tissue elastance of these animals [187]. Park et al. also demonstrated that the human tripeptide glycyl-l-histidyl-l-lysine reduced the reactive oxygen species, TNF-α, and IL-6 production, in murine macrophages stimulated with LPS [190]. These data suggest that this tripeptide is relevant for controlling inflammation and preventing lung remodeling, at least in animals.

Although lung remodeling in ARDS is still not fully understood, lung repair and remodeling, including all the alterations discussed above, is necessary, to allow for the recovery of ARDS. In this regard, Pinheiro et al. clearly showed that pharmacological stimulation of nicotinic receptors by PNU changed macrophage profile from M1 through M2 subtypes. This treatment also attenuated collagen deposition, suggesting that, this change in macrophage profile can explain the resolution of lung inflammation and the improvement in lung function observed in this study [191]. Table 4 summarizes lung function changes and markers of ECM remodeling, in clinical and experimental studies of ARDS.

### 4.3. Final Considerations

Structural changes in ECM components are associated with the worsening of lung function and the progression of asthma, COPD, and ARDS. In this context, clinical studies have been useful for characterizing which ECM components are present in the lung samples of patients, mainly those in advanced stages of respiratory diseases, and for investigating the associations between these changes and the progression of these diseases. In addition, some ECM proteins and inflammatory mediator markers in the serum of patients, have been used as important features for elucidating the extent of structural changes in the lungs, to avoid invasive procedures, facilitating a prognostic evaluation of these respiratory diseases.

In animal models, temporal analyses of inflammatory profiles and respiratory function have been performed, to elucidate the different mechanisms involved in disease progression. Moreover, the opportunity to evaluate in vivo responses to treatment, with inhibitors of specific inflammatory mediators, has better elucidated the different mechanisms involved in the pathogenesis of these diseases and highlighted some possible therapeutic targets, since most of these inhibitors have been shown to attenuate fiber remodeling and improve lung function. It is important to note that although many experimental models have been described in the literature for inducing different respiratory diseases, none of them recreates all of the physiological changes observed in humans. Therefore, before choosing an experimental model, it is very important to consider which inflammatory events and structural changes can be evaluated with different approaches.

In vitro studies [46,47,87,92,125,128,129,153,167,186,189,190,192] have improved our understanding of which ECM components change and elucidate their effects on the impairment of respiratory parameters; they have also made it possible to analyze how different inflammatory mediators impact inflammatory cell activity and recruitment.

However, these studies have some limitations. Despite these advances in our understanding of the mechanisms involved in ECM structural changes in asthma, COPD, and ARDS, there are no clinical studies that have showed an effective treatment to reverse all structural changes, in order to totally restore the lung function. Bronchodilators and corticosteroids have been used to relieve the symptoms of these respiratory diseases, but these approaches cannot control disease progression.

Further investigations are necessary to distinguish how the dysregulation of the different ECM components drive these structural changes progression, as well as how the interactions between cells and the ECM components, during these disease progressions, could impact the lung function.

## Figures and Tables

**Table 1:** Lung function changes and markers of airway remodeling in asthma.

	Clinical Studies	Experimental Studies *
**Lung Function**		
	Moderate to severe stages of asthma (FEV1 < 80%) [42,52,66,67]	↑ in lung tissue resistance and elastance [48,58,59,60,69,88,89]
**Remodeling Markers**		
Fibroblast	↑ in airways (severe stage) [52]	—
Collagen fibers	—	↑ in airways [48,58,59,69,88] ↑ in lung parenchyma [58,59,60,69,88,89]
Collagen subtype I	↑ in airways (moderate and severe stages) [42]	↑ in lung parenchyma [57]
Collagen subtype III	↑ in airways (severe stage) [52] ↑ in airways (moderate and severe stages) [42]	↑ in lung parenchyma [57]
Elastic fibers	↑ in large ASM in fatal asthma (severe stage) [66]	↑ in airways [48,59,69] ↑ in lung parenchyma [59,60]
Decorin	—	↑ in airways [58] ↑ in lung parenchyma [57,58]
Lumican	↑ in subepithelial layer (severe stage) [67] ↑ in ASM (moderate stage) [67]	↑ in lung parenchyma [57]
Actin	—	↑ in airways [69] ↑ in lung parenchyma [57,60,69]
Biglycan	↑ in ASM (moderate stage) [67]	↑ in airways [58] ↑ in lung parenchyma [57,58]
Fibronectin	↑ in large ASM in fatal asthma (severe stage) [66]	↑ in airways [58] ↑ in lung parenchyma [57,58]

FEV_1_: Forced expiratory volume in 1 s; ASM: Airway smooth muscle. * OVA-induced asthma model.

**Table 2:** Classification of airflow limitation severity in chronic obstructive pulmonary disease (COPD).

In Patients with FEV_1_/FVC < 0.70:		
GOLD 1	Mild	FEV_1_ ≥ 80% predicted
GOLD 2	Moderate	50% ≤ FEV_1_ < 80% predicted
GOLD 3	Severe	30% ≤ FEV_1_ < 50% predicted
GOLD 4	Very Severe	FEV_1_ < 30% predicted

GOLD: Global Initiative for Chronic Obstructive Lung Disease; FEV_1_: Forced expiratory volume in 1 s; FVC: Forced vital capacity.

**Table 3:** Lung function changes and markers of extracellular matrix (ECM) remodeling in COPD.

	Clinical Studies	Experimental Studies *
**Lung Function**		
	Mild to severe stages of COPD [5,22,112,122,128,129] (FEV1/FVC < 70%, FEV1 ≥ 80%)	↓ in lung tissue elastance and resistance [115,143,146,154]
**Remodeling Markers**		
Collagen fibers	↑ in SEBM (moderate and severe stages) [10] ↑ in small airways (moderate and severe stages) [128] ↑ in interstitial matrix (mild to moderate stages) [112]	↑ in lung parenchyma [115,143,146,154]
Collagen subtype I	↑ in SEBM, lamina propria and bronchial adventitia (moderate and severe stages) [22] ↑ in airway (moderate stage) [129] ↓ in small and large airways (moderate stage) [5]	↓ in lung parenchyma during COPD development [150]
Collagen subtype Ill	↑ in SEBM, lamina propria and bronchial adventitia (moderate and severe stages) [22]	↑ in lung parenchyma [145,150] ↓ in lung parenchyma during COPD development [150]
Collagen subtype IV	↑ in SEBM (moderate and severe stages) [22]	—
Elastic fibers	↓ in alveoli and small airways (moderate stage) [122]	↑ in lung parenchyma [115,143,146] ↓ in lung parenchyma and basement membrane [117]
Elastin	↑ in interstitial matrix (mild to moderate stages) [112]	↑ in lung parenchyma [145,150] ↓ in lung parenchyma during COPD development [150]
Fibrillin	—	↑ in lung parenchyma [145]
Fibronectin	↑ in SEBM (moderate and severe stages) [22] ↑ in small airways (moderate stage) [5]	—

ECM: Extracellular matrix; COPD: Chronic obstructive pulmonary disease; FEV_1_: Forced expiratory volume in 1 s; FVC: Forced vital capacity; SEBM: Surface epithelial basement membrane; PPE: Porcine pancreatic elastase. * COPD model induced by PPE [117,145,146,150] or papain instillation [112], or cigarette smoke exposure [143,145,154].

**Table 4:** Lung function changes and markers of ECM remodeling in acute respiratory distress syndrome (ARDS).

	Clinical Studies	Experimental Studies *
**Lung Function**		
	Moderate to severe stage of ARDS (PaO2/FIO2 ≤ 200 mmHg) [179,180,181]	↑ in respiratory system resistance after 24 h [174,191] ↑ in respiratory system elastance after 24 h [174,187,191] ↑ in lung tissue resistance after 24 h [191] ↑ in lung tissue elastance after 24 h [187,191]
**Remodeling Markers**		
Collagen fibers	↑ in lung parenchyma (late phase) [172]	↑ in lung parenchyma after 24 h [174,187,191] ↑ in lung parenchyma after 5 weeks [188]
Collagen subtype I	↑ in BALF and serum (severe stage) [180]	↑ in lung parenchyma after 5 weeks [188]
Collagen subtype III	↑ in BALF and serum (severe stage) [180]	—
Elastic fibers	↑ in lung parenchyma (late phase) [172]	↑ in lung parenchyma after 1 week [174]
Procollagen type I	↑ in plasma (moderate and severe stages) [181]	—
Procollagen type III	↑ in BALF (severe stage) [179,180] ↑ in serum (severe stage) [180] ↑ in plasma (moderate and severe stages) [181]	—

ECM: Extracellular matrix; ARDS: Acute respiratory distress syndrome; PaO2/FIO2: Arterial partial pressure of oxygen/fraction of inspired oxygen; ALI: Acute lung injury; BALF: Bronchoalveolar lavage fluid; LPS: Lipopolysaccharide. * ALI induced by Escherichia coli LPS intratracheal or intraperitoneal administration [174]. ALI induced by LPS intranasal [187] or intratracheal administration [191] or LPS nebulization [188].

## References

[1] Suki B., Bates J.H. (2008). Extracellular matrix mechanics in lung parenchymal diseases. Respir. Physiol. Neurobiol..

[2] Burgstaller G., Oehrle B., Gerckens M., White E.S., Schiller H.B., Eickelberg O. (2017). The instructive extracellular matrix of the lung: Basic composition and alterations in chronic lung disease. Eur. Respir. J..

[3] Manuyakorn W., Howarth P.H., Holgate S.T. (2013). Airway remodelling in asthma and novel therapy. Asian Pac. J. Allergy Immunol..

[4] Yue B. (2014). Biology of the extracellular matrix: An overview. J. Glaucoma.

[5] Annoni R., Lancas T., Yukimatsu Tanigawa R., de Medeiros Matsushita M., de Morais Fernezlian S., Bruno A., Fernando Ferraz da Silva L., Roughley P.J., Battaglia S., Dolhnikoff M. (2012). Extracellular matrix composition in COPD. Eur. Respir. J..

[6] Silver F.H., Birk D.E. (1984). Molecular structure of collagen in solution: Comparison of types I, II, III and V. Int. J. Biol. Macromol..

[7] Shifren A., Mecham R.P. (2006). The stumbling block in lung repair of emphysema: Elastic fiber assembly. Proc. Am. Thorac. Soc..

[8] Robbesom A.A., Koenders M.M., Smits N.C., Hafmans T., Versteeg E.M., Bulten J., Veerkamp J.H., Dekhuijzen P.N., van Kuppevelt T.H. (2008). Aberrant fibrillin-1 expression in early emphysematous human lung: A proposed predisposition for emphysema. Mod. Pathol..

[9] Brown R.E., Butler J.P., Rogers R.A., Leith D.E. (1994). Mechanical connections between elastin and collagen. Connect. Tissue Res..

[10] Raspanti M., Alessandrini A., Ottani V., Ruggeri A. (1997). Direct visualization of collagen-bound proteoglycans by tapping-mode atomic force microscopy. J. Struct. Biol..

[11] Kielty C.M., Sherratt M.J., Shuttleworth C.A. (2002). Elastic fibres. J. Cell Sci..

[12] Setnikar I. (1955). Origin and significance of the mechanical property of the lung. Arch. Fisiol..

[13] Yuan H., Kononov S., Cavalcante F.S., Lutchen K.R., Ingenito E.P., Suki B. (2000). Effects of collagenase and elastase on the mechanical properties of lung tissue strips. J. Appl. Physiol..

[14] Lever R., Page C. (2001). Glycosaminoglycans, airways inflammation and bronchial hyperresponsiveness. Pulm. Pharmacol. Ther..

[15] Tyrrell D.J., Horne A.P., Holme K.R., Preuss J.M., Page C.P. (1999). Heparin in inflammation: Potential therapeutic applications beyond anticoagulation. Adv. Pharmacol..

[16] Salazar L.M., Herrera A.M. (2011). Fibrotic response of tissue remodeling in COPD. Lung.

[17] Malmstrom J., Larsen K., Malmstrom L., Tufvesson E., Parker K., Marchese J., Williamson B., Hattan S., Patterson D., Martin S. (2004). Proteome annotations and identifications of the human pulmonary fibroblast. J. Proteome Res..

[18] Zandvoort A., Postma D.S., Jonker M.R., Noordhoek J.A., Vos J.T., Timens W. (2008). Smad gene expression in pulmonary fibroblasts: Indications for defective ECM repair in COPD. Respir. Res..

[19] Hallgren O., Nihlberg K., Dahlback M., Bjermer L., Eriksson L.T., Erjefalt J.S., Lofdahl C.G., Westergren-Thorsson G. (2010). Altered fibroblast proteoglycan production in COPD. Respir. Res..

[20] Hill M.R., Philp C.J., Billington C.K., Tatler A.L., Johnson S.R., O’Dea R.D., Brook B.S. (2018). A theoretical model of inflammation- and mechanotransduction-driven asthmatic airway remodelling. Biomech. Model. Mechanobiol..

[21] Zandvoort A., Postma D.S., Jonker M.R., Noordhoek J.A., Vos J.T., van der Geld Y.M., Timens W. (2006). Altered expression of the Smad signalling pathway: Implications for COPD pathogenesis. Eur. Respir. J..

[22] Kranenburg A.R., Willems-Widyastuti A., Moori W.J., Sterk P.J., Alagappan V.K., de Boer W.I., Sharma H.S. (2006). Enhanced bronchial expression of extracellular matrix proteins in chronic obstructive pulmonary disease. Am. J. Clin. Pathol..

[23] Loftus P.A., Wise S.K. (2016). Epidemiology of asthma. Curr. Opin. Otolaryngol. Head Neck Surg..

[24] Moorman J.E., Akinbami L.J., Bailey C.M., Johnson C.A., King M.E., Liu X., Zahran H.S. (2012). National surveillance of asthma: United States, 2001–2010. Vital Health Stat. Ser. Anal. Epidemiol. Stud..

[25] Global Initiative for Asthma (GINA) (2018). Global Strategy for Asthma Management and Prevention. https://ginasthma.org/.

[26] Branchett W.J., Lloyd C.M. (2019). Regulatory cytokine function in the respiratory tract. Mucosal Immunol..

[27] Ubel C., Graser A., Koch S., Rieker R.J., Lehr H.A., Muller M., Finotto S. (2014). Role of Tyk-2 in Th9 and Th17 cells in allergic asthma. Sci. Rep..

[28] Peng J., Li X.M., Zhang G.R., Cheng Y., Chen X., Gu W., Guo X.J. (2019). TNF-TNFR2 Signaling Inhibits Th2 and Th17 Polarization and Alleviates Allergic Airway Inflammation. Int. Arch. Allergy Immunol..

[29] Al-Muhsen S., Johnson J.R., Hamid Q. (2011). Remodeling in asthma. J. Allergy Clin. Immunol..

[30] Nishimura Y., Inoue T., Morooka T., Node K. (2008). Mechanical stretch and angiotensin II increase interleukin-13 production and interleukin-13 receptor alpha2 expression in rat neonatal cardiomyocytes. Circ. J..

[31] Tschumperlin D.J., Drazen J.M. (2001). Mechanical stimuli to airway remodeling. Am. J. Respir. Crit. Care Med..

[32] Miyagawa A., Chiba M., Hayashi H., Igarashi K. (2009). Compressive force induces VEGF production in periodontal tissues. J. Dent. Res..

[33] Park J.A., Tschumperlin D.J. (2009). Chronic intermittent mechanical stress increases MUC5AC protein expression. Am. J. Respir. Cell Mol. Biol..

[34] Park J.A., Drazen J.M., Tschumperlin D.J. (2010). The chitinase-like protein YKL-40 is secreted by airway epithelial cells at base line and in response to compressive mechanical stress. J. Biol. Chem..

[35] Grainge C.L., Lau L.C., Ward J.A., Dulay V., Lahiff G., Wilson S., Holgate S., Davies D.E., Howarth P.H. (2011). Effect of bronchoconstriction on airway remodeling in asthma. N. Engl. J. Med..

[36] Pohunek P., Warner J.O., Turzikova J., Kudrmann J., Roche W.R. (2005). Markers of eosinophilic inflammation and tissue re-modelling in children before clinically diagnosed bronchial asthma. Pediatr. Allergy Immunol..

[37] Lezmi G., Gosset P., Deschildre A., Abou-Taam R., Mahut B., Beydon N., de Blic J. (2015). Airway Remodeling in Preschool Children with Severe Recurrent Wheeze. Am. J. Respir. Crit. Care Med..

[38] James A. (2005). Airway remodeling in asthma. Curr. Opin. Pulm. Med..

[39] Lazaar A.L., Panettieri R.A. (2003). Is airway remodeling clinically relevant in asthma?. Am. J. Med..

[40] Cohn L., Elias J.A., Chupp G.L. (2004). Asthma: Mechanisms of disease persistence and progression. Annu. Rev. Immunol..

[41] James A. (2017). Airway Remodeling in Asthma: Is it Fixed or Variable?. Am. J. Respir. Crit. Care Med..

[42] Chakir J., Shannon J., Molet S., Fukakusa M., Elias J., Laviolette M., Boulet L.P., Hamid Q. (2003). Airway remodeling-associated mediators in moderate to severe asthma: Effect of steroids on TGF-beta, IL-11, IL-17, and type I and type III collagen expression. J. Allergy Clin. Immunol..

[43] Kaminska M., Foley S., Maghni K., Storness-Bliss C., Coxson H., Ghezzo H., Lemiere C., Olivenstein R., Ernst P., Hamid Q. (2009). Airway remodeling in subjects with severe asthma with or without chronic persistent airflow obstruction. J. Allergy Clin. Immunol..

[44] Slats A.M., Janssen K., van Schadewijk A., van der Plas D.T., Schot R., van den Aardweg J.G., de Jongste J.C., Hiemstra P.S., Mauad T., Rabe K.F. (2008). Expression of smooth muscle and extracellular matrix proteins in relation to airway function in asthma. J. Allergy Clin. Immunol..

[45] Yick C.Y., Ferreira D.S., Annoni R., von der Thusen J.H., Kunst P.W., Bel E.H., Lutter R., Mauad T., Sterk P.J. (2012). Extracellular matrix in airway smooth muscle is associated with dynamics of airway function in asthma. Allergy.

[46] Khan M.A., Kianpour S., Stampfli M.R., Janssen L.J. (2007). Kinetics of in vitro bronchoconstriction in an elastolytic mouse model of emphysema. Eur. Respir. J..

[47] Khan M.A., Ellis R., Inman M.D., Bates J.H., Sanderson M.J., Janssen L.J. (2010). Influence of airway wall stiffness and parenchymal tethering on the dynamics of bronchoconstriction. Am. J. Physiol. Lung Cell. Mol. Physiol..

[48] Possa S.S., Charafeddine H.T., Righetti R.F., da Silva P.A., Almeida-Reis R., Saraiva-Romanholo B.M., Perini A., Prado C.M., Leick-Maldonado E.A., Martins M.A. (2012). Rho-kinase inhibition attenuates airway responsiveness, inflammation, matrix remodeling, and oxidative stress activation induced by chronic inflammation. Am. J. Physiol. Lung Cell. Mol. Physiol..

[49] Joubert P., Hamid Q. (2005). Role of airway smooth muscle in airway remodeling. J. Allergy Clin. Immunol..

[50] James A.L., Pare P.D., Hogg J.C. (1989). The mechanics of airway narrowing in asthma. Am. Rev. Respir. Dis..

[51] Holloway L., Beasley R., Roche W., Busse W., Holgate S. (1995). The pathology of bronchial asthma. Asthma and Rhinitis.

[52] Benayoun L., Druilhe A., Dombret M.C., Aubier M., Pretolani M. (2003). Airway structural alterations selectively associated with severe asthma. Am. J. Respir. Crit. Care Med..

[53] Keglowich L.F., Borger P. (2015). The Three A’s in Asthma—Airway Smooth Muscle, Airway Remodeling & Angiogenesis. Open Respir. Med. J..

[54] Kotaru C., Schoonover K.J., Trudeau J.B., Huynh M.L., Zhou X., Hu H., Wenzel S.E. (2006). Regional fibroblast heterogeneity in the lung: Implications for remodeling. Am. J. Respir. Crit. Care Med..

[55] Ojiaku C.A., Yoo E.J., Panettieri R.A. (2017). Transforming Growth Factor beta1 Function in Airway Remodeling and Hyperresponsiveness. The Missing Link?. Am. J. Respir. Cell Mol. Biol..

[56] Burgess J.K., Mauad T., Tjin G., Karlsson J.C., Westergren-Thorsson G. (2016). The extracellular matrix—The under-recognized element in lung disease?. J. Pathol..

[57] Camargo L.D.N., Righetti R.F., Aristoteles L., Dos Santos T.M., de Souza F.C.R., Fukuzaki S., Cruz M.M., Alonso-Vale M.I.C., Saraiva-Romanholo B.M., Prado C.M. (2017). Effects of Anti-IL-17 on Inflammation, Remodeling, and Oxidative Stress in an Experimental Model of Asthma Exacerbated by LPS. Front. Immunol..

[58] Dos Santos T.M., Righetti R.F., Camargo L.D.N., Saraiva-Romanholo B.M., Aristoteles L., de Souza F.C.R., Fukuzaki S., Alonso-Vale M.I.C., Cruz M.M., Prado C.M. (2018). Effect of Anti-IL17 Antibody Treatment Alone and in Combination With Rho-Kinase Inhibitor in a Murine Model of Asthma. Front. Physiol..

[59] Bortolozzo A.S.S., Rodrigues A.P.D., Arantes-Costa F.M., Saraiva-Romanholo B.M., de Souza F.C.R., Bruggemann T.R., de Brito M.V., Ferreira R.D.S., Correia M., Paiva P.M.G. (2018). The Plant Proteinase Inhibitor CrataBL Plays a Role in Controlling Asthma Response in Mice. BioMed Res. Int..

[60] Righetti R.F., Pigati P.A., Possa S.S., Habrum F.C., Xisto D.G., Antunes M.A., Leick E.A., Prado C.M., Martins Mde A., Rocco P.R. (2014). Effects of Rho-kinase inhibition in lung tissue with chronic inflammation. Respir. Physiol. Neurobiol..

[61] Reese S.P., Underwood C.J., Weiss J.A. (2013). Effects of decorin proteoglycan on fibrillogenesis, ultrastructure, and mechanics of type I collagen gels. Matrix Biol..

[62] Kalamajski S., Oldberg A. (2010). The role of small leucine-rich proteoglycans in collagen fibrillogenesis. Matrix Biol..

[63] Sarrazin S., Lamanna W.C., Esko J.D. (2011). Heparan sulfate proteoglycans. Cold Spring Harb. Perspect. Biol..

[64] Chen H.C., Chang H.T., Huang P.H., Chang M.D., Liu R.S., Lin Y.J., Hsieh C.H. (2013). Molecular imaging of heparan sulfate expression with radiolabeled recombinant eosinophil cationic protein predicts allergic lung inflammation in a mouse model for asthma. J. Nucl. Med..

[65] Tanaka Y., Adams D.H., Shaw S. (1993). Proteoglycans on endothelial cells present adhesion-inducing cytokines to leukocytes. Immunol. Today.

[66] Araujo B.B., Dolhnikoff M., Silva L.F., Elliot J., Lindeman J.H., Ferreira D.S., Mulder A., Gomes H.A., Fernezlian S.M., James A. (2008). Extracellular matrix components and regulators in the airway smooth muscle in asthma. Eur. Respir. J..

[67] Pini L., Hamid Q., Shannon J., Lemelin L., Olivenstein R., Ernst P., Lemiere C., Martin J.G., Ludwig M.S. (2007). Differences in proteoglycan deposition in the airways of moderate and severe asthmatics. Eur. Respir. J..

[68] Murphy G. (2011). Tissue inhibitors of metalloproteinases. Genome Biol..

[69] Pigati P.A., Righetti R.F., Possa S.S., Romanholo B.S., Rodrigues A.P., dos Santos A.S., Xisto D.G., Antunes M.A., Prado C.M., Leick E.A. (2015). Y-27632 is associated with corticosteroid-potentiated control of pulmonary remodeling and inflammation in guinea pigs with chronic allergic inflammation. BMC Pulm. Med..

[70] James A.L., Wenzel S. (2007). Clinical relevance of airway remodelling in airway diseases. Eur. Respir. J..

[71] Ward C., Pais M., Bish R., Reid D., Feltis B., Johns D., Walters E.H. (2002). Airway inflammation, basement membrane thickening and bronchial hyperresponsiveness in asthma. Thorax.

[72] Chetta A., Zanini A., Foresi A., Del Donno M., Castagnaro A., D’Ippolito R., Baraldo S., Testi R., Saetta M., Olivieri D. (2003). Vascular component of airway remodeling in asthma is reduced by high dose of fluticasone. Am. J. Respir. Crit. Care Med..

[73] Laitinen L.A., Laitinen A., Haahtela T. (1992). A comparative study of the effects of an inhaled corticosteroid, budesonide, and a beta 2-agonist, terbutaline, on airway inflammation in newly diagnosed asthma: A randomized, double-blind, parallel-group controlled trial. J. Allergy Clin. Immunol..

[74] Hoshino M., Takahashi M., Takai Y., Sim J., Aoike N. (2001). Inhaled corticosteroids decrease vascularity of the bronchial mucosa in patients with asthma. Clin. Exp. Allergy.

[75] Sont J.K., Han J., van Krieken J.M., Evertse C.E., Hooijer R., Willems L.N., Sterk P.J. (1996). Relationship between the inflammatory infiltrate in bronchial biopsy specimens and clinical severity of asthma in patients treated with inhaled steroids. Thorax.

[76] Elliot J.G., Noble P.B., Mauad T., Bai T.R., Abramson M.J., McKay K.O., Green F.H.Y., James A.L. (2018). Inflammation-dependent and independent airway remodelling in asthma. Respirology.

[77] Berair R., Brightling C.E. (2014). Asthma therapy and its effect on airway remodelling. Drugs.

[78] Pretolani M., Dombret M.C., Thabut G., Knap D., Hamidi F., Debray M.P., Taille C., Chanez P., Aubier M. (2014). Reduction of airway smooth muscle mass by bronchial thermoplasty in patients with severe asthma. Am. J. Respir. Crit. Care Med..

[79] Chakir J., Haj-Salem I., Gras D., Joubert P., Beaudoin E.L., Biardel S., Lampron N., Martel S., Chanez P., Boulet L.P. (2015). Effects of Bronchial Thermoplasty on Airway Smooth Muscle and Collagen Deposition in Asthma. Ann. Am. Thorac. Soc..

[80] Williams K., Roman J. (2016). Studying human respiratory disease in animals--role of induced and naturally occurring models. J. Pathol..

[81] Zosky G.R., Sly P.D. (2007). Animal models of asthma. Clin. Exp. Allergy.

[82] Mullane K., Williams M. (2014). Animal models of asthma: Reprise or reboot?. Biochem. Pharmacol..

[83] Shin Y.S., Takeda K., Gelfand E.W. (2009). Understanding asthma using animal models. Allergy Asthma Immunol. Res..

[84] Aun M.V., Bonamichi-Santos R., Arantes-Costa F.M., Kalil J., Giavina-Bianchi P. (2017). Animal models of asthma: Utility and limitations. J. Asthma Allergy.

[85] Almeida-Reis R., Toledo A.C., Reis F.G., Marques R.H., Prado C.M., Dolhnikoff M., Martins M.A., Leick-Maldonado E.A., Tiberio I.F. (2010). Repeated stress reduces mucociliary clearance in animals with chronic allergic airway inflammation. Respir. Physiol. Neurobiol..

[86] Bates J.H., Davis G.S., Majumdar A., Butnor K.J., Suki B. (2007). Linking parenchymal disease progression to changes in lung mechanical function by percolation. Am. J. Respir. Crit. Care Med..

[87] Park G.M., Han H.W., Kim J.Y., Lee E., Cho H.J., Yoon J., Hong S.J., Yang S.I., Yang H.J., Yu J. (2016). Association of symptom control with changes in lung function, bronchial hyperresponsiveness, and exhaled nitric oxide after inhaled corticosteroid treatment in children with asthma. Allergol. Int..

[88] Abreu S.C., Antunes M.A., Mendonca L., Branco V.C., de Melo E.B., Olsen P.C., Diaz B.L., Weiss D.J., Paredes B.D., Xisto D.G. (2014). Effects of bone marrow mononuclear cells from healthy or ovalbumin-induced lung inflammation donors on recipient allergic asthma mice. Stem Cell Res. Ther..

[89] Marques R.H., Reis F.G., Starling C.M., Cabido C., de Almeida-Reis R., Dohlnikoff M., Prado C.M., Leick E.A., Martins M.A., Tiberio I.F. (2012). Inducible nitric oxide synthase inhibition attenuates physical stress-induced lung hyper-responsiveness and oxidative stress in animals with lung inflammation. Neuroimmunomodulation.

[90] Vasconcelos L.H.C., Silva M., Costa A.C., de Oliveira G.A., de Souza I.L.L., Queiroga F.R., Araujo L., Cardoso G.A., Righetti R.F., Silva A.S. (2018). A Guinea Pig Model of Airway Smooth Muscle Hyperreactivity Induced by Chronic Allergic Lung Inflammation: Contribution of Epithelium and Oxidative Stress. Front. Pharmacol..

[91] Hallstrand T.S., Henderson W.R. (2010). An update on the role of leukotrienes in asthma. Curr. Opin. Allergy Clin. Immunol..

[92] Pardo-Saganta A., Law B.M., Gonzalez-Celeiro M., Vinarsky V., Rajagopal J. (2013). Ciliated cells of pseudostratified airway epithelium do not become mucous cells after ovalbumin challenge. Am. J. Respir. Cell Mol. Biol..

[93] Evans C.M., Koo J.S. (2009). Airway mucus: The good, the bad, the sticky. Pharmacol. Ther..

[94] Bonser L.R., Erle D.J. (2017). Airway Mucus and Asthma: The Role of MUC5AC and MUC5B. J. Clin. Med..

[95] Lancas T., Kasahara D.I., Prado C.M., Tiberio I.F., Martins M.A., Dolhnikoff M. (2006). Comparison of early and late responses to antigen of sensitized guinea pig parenchymal lung strips. J. Appl. Physiol..

[96] van der Worp H.B., Howells D.W., Sena E.S., Porritt M.J., Rewell S., O’Collins V., Macleod M.R. (2010). Can animal models of disease reliably inform human studies?. PLoS Med..

[97] Global Initiative for Chronic Obstructive Lung Disease (2019). Global Strategy for the Diagnosis, Management and Prevention of COPD 2019.

[98] Brandsma C.A., Timens W., Jonker M.R., Rutgers B., Noordhoek J.A., Postma D.S. (2013). Differential effects of fluticasone on extracellular matrix production by airway and parenchymal fibroblasts in severe COPD. Am. J. Physiol. Lung Cell. Mol. Physiol..

[99] Barnes P.J., Stockley R.A. (2005). COPD: Current therapeutic interventions and future approaches. Eur. Respir. J..

[100] Brusselle G.G., Joos G.F., Bracke K.R. (2011). New insights into the immunology of chronic obstructive pulmonary disease. Lancet.

[101] Higham A., Quinn A.M., Cancado J.E.D., Singh D. (2019). The pathology of small airways disease in COPD: Historical aspects and future directions. Respir. Res..

[102] Hogg J.C., Timens W. (2009). The pathology of chronic obstructive pulmonary disease. Annu. Rev. Pathol..

[103] Imai K., Dalal S.S., Chen E.S., Downey R., Schulman L.L., Ginsburg M., D’Armiento J. (2001). Human collagenase (matrix metalloproteinase-1) expression in the lungs of patients with emphysema. Am. J. Respir. Crit. Care Med..

[104] Segura-Valdez L., Pardo A., Gaxiola M., Uhal B.D., Becerril C., Selman M. (2000). Upregulation of gelatinases A and B, collagenases 1 and 2, and increased parenchymal cell death in COPD. Chest.

[105] Churg A., Wang R., Wang X., Onnervik P.O., Thim K., Wright J.L. (2007). Effect of an MMP-9/MMP-12 inhibitor on smoke-induced emphysema and airway remodelling in guinea pigs. Thorax.

[106] Churg A., Zhou S., Wright J.L. (2012). Series “matrix metalloproteinases in lung health and disease”: Matrix metalloproteinases in COPD. Eur. Respir. J..

[107] Hautamaki R.D., Kobayashi D.K., Senior R.M., Shapiro S.D. (1997). Requirement for macrophage elastase for cigarette smoke-induced emphysema in mice. Science.

[108] Abraham T., Hogg J. (2010). Extracellular matrix remodeling of lung alveolar walls in three dimensional space identified using second harmonic generation and multiphoton excitation fluorescence. J. Struct. Biol..

[109] Shifren A., Durmowicz A.G., Knutsen R.H., Hirano E., Mecham R.P. (2007). Elastin protein levels are a vital modifier affecting normal lung development and susceptibility to emphysema. Am. J. Physiol. Lung Cell. Mol. Physiol..

[110] Kononov S., Brewer K., Sakai H., Cavalcante F.S., Sabayanagam C.R., Ingenito E.P., Suki B. (2001). Roles of mechanical forces and collagen failure in the development of elastase-induced emphysema. Am. J. Respir. Crit. Care Med..

[111] Koenders M.M., Wismans R.G., Starcher B., Hamel B.C., Dekhuijzen R.P., van Kuppevelt T.H. (2009). Fibrillin-1 staining anomalies are associated with increased staining for TGF-beta and elastic fibre degradation; new clues to the pathogenesis of emphysema. J. Pathol..

[112] Vlahovic G., Russell M.L., Mercer R.R., Crapo J.D. (1999). Cellular and connective tissue changes in alveolar septal walls in emphysema. Am. J. Respir. Crit. Care Med..

[113] Rubio M.L., Martin-Mosquero M.C., Ortega M., Peces-Barba G., Gonzalez-Mangado N. (2004). Oral N-acetylcysteine attenuates elastase-induced pulmonary emphysema in rats. Chest.

[114] Churg A., Wang R.D., Tai H., Wang X., Xie C., Wright J.L. (2004). Tumor necrosis factor-alpha drives 70% of cigarette smoke-induced emphysema in the mouse. Am. J. Respir. Crit. Care Med..

[115] Anciaes A.M., Olivo C.R., Prado C.M., Kagohara K.H., Pinto Tda S., Moriya H.T., Mauad T., Martins Mde A., Lopes F.D. (2011). Respiratory mechanics do not always mirror pulmonary histological changes in emphysema. Clinics.

[116] Stockley R.A. (2001). Proteases and antiproteases. Novartis Found. Symp..

[117] Lucey E.C., Goldstein R.H., Stone P.J., Snider G.L. (1998). Remodeling of alveolar walls after elastase treatment of hamsters. Results of elastin and collagen mRNA in situ hybridization. Am. J. Respir. Crit. Care Med..

[118] Kawakami M., Matsuo Y., Yoshiura K., Nagase T., Yamashita N. (2008). Sequential and quantitative analysis of a murine model of elastase-induced emphysema. Biol. Pharm. Bull..

[119] Parameswaran K., Willems-Widyastuti A., Alagappan V.K., Radford K., Kranenburg A.R., Sharma H.S. (2006). Role of extracellular matrix and its regulators in human airway smooth muscle biology. Cell Biochem. Biophys..

[120] Li H., Cui D., Ma N., Lu L., Gao Y., Cui X., Wang D. (2002). The effect of extracellular matrix remodeling on airflow obstruction in a rat model of chronic obstructive pulmonary disease. Zhonghua Jie He He Hu Xi Za Zhi.

[121] Bidan C.M., Veldsink A.C., Meurs H., Gosens R. (2015). Airway and Extracellular Matrix Mechanics in COPD. Front. Physiol..

[122] Black P.N., Ching P.S., Beaumont B., Ranasinghe S., Taylor G., Merrilees M.J. (2008). Changes in elastic fibres in the small airways and alveoli in COPD. Eur. Respir. J..

[123] Gladysheva E.S., Malhotra A., Owens R.L. (2010). Influencing the decline of lung function in COPD: Use of pharmacotherapy. Int. J. Chronic Obstr. Pulm. Dis..

[124] Pare P.D., Mitzner W. (2012). Airway-parenchymal interdependence. Compr. Physiol..

[125] Chen L., Ge Q., Tjin G., Alkhouri H., Deng L., Brandsma C.A., Adcock I., Timens W., Postma D., Burgess J.K. (2014). Effects of cigarette smoke extract on human airway smooth muscle cells in COPD. Eur. Respir. J..

[126] Hogg J.C., Chu F., Utokaparch S., Woods R., Elliott W.M., Buzatu L., Cherniack R.M., Rogers R.M., Sciurba F.C., Coxson H.O. (2004). The nature of small-airway obstruction in chronic obstructive pulmonary disease. N. Engl. J. Med..

[127] Eurlings I.M., Dentener M.A., Cleutjens J.P., Peutz C.J., Rohde G.G., Wouters E.F., Reynaert N.L. (2014). Similar matrix alterations in alveolar and small airway walls of COPD patients. BMC Pulm. Med..

[128] Sun C., Zhu M., Yang Z., Pan X., Zhang Y., Wang Q., Xiao W. (2014). LL-37 secreted by epithelium promotes fibroblast collagen production: A potential mechanism of small airway remodeling in chronic obstructive pulmonary disease. Lab. Investig..

[129] Milara J., Peiro T., Serrano A., Cortijo J. (2013). Epithelial to mesenchymal transition is increased in patients with COPD and induced by cigarette smoke. Thorax.

[130] Mahmood M.Q., Reid D., Ward C., Muller H.K., Knight D.A., Sohal S.S., Walters E.H. (2017). Transforming growth factor (TGF) beta1 and Smad signalling pathways: A likely key to EMT-associated COPD pathogenesis. Respirology.

[131] Podowski M., Calvi C., Metzger S., Misono K., Poonyagariyagorn H., Lopez-Mercado A., Ku T., Lauer T., McGrath-Morrow S., Berger A. (2012). Angiotensin receptor blockade attenuates cigarette smoke-induced lung injury and rescues lung architecture in mice. J. Clin. Investig..

[132] Wang Z., Fang K., Wang G., Guan X., Pang Z., Guo Y., Yuan Y., Ran N., Liu Y., Wang F. (2019). Protective effect of amygdalin on epithelial-mesenchymal transformation in experimental chronic obstructive pulmonary disease mice. Phytother. Res..

[133] Sand J.M., Leeming D.J., Byrjalsen I., Bihlet A.R., Lange P., Tal-Singer R., Miller B.E., Karsdal M.A., Vestbo J. (2016). High levels of biomarkers of collagen remodeling are associated with increased mortality in COPD—Results from the ECLIPSE study. Respir. Res..

[134] Zeng Y.Y., Hu W.P., Zuo Y.H., Wang X.R., Zhang J. (2019). Altered serum levels of type I collagen turnover indicators accompanied by IL-6 and IL-8 release in stable COPD. Int. J. Chronic Obstr. Pulm. Dis..

[135] Vignola A.M., Paganin F., Capieu L., Scichilone N., Bellia M., Maakel L., Bellia V., Godard P., Bousquet J., Chanez P. (2004). Airway remodelling assessed by sputum and high-resolution computed tomography in asthma and COPD. Eur. Respir. J..

[136] Papakonstantinou E., Bonovolias I., Roth M., Tamm M., Schumann D., Baty F., Louis R., Milenkovic B., Boersma W., Stieltjes B. (2019). Serum levels of hyaluronic acid are associated with COPD severity and predict survival. Eur. Respir. J..

[137] Suki B., Lutchen K.R., Ingenito E.P. (2003). On the progressive nature of emphysema: Roles of proteases, inflammation, and mechanical forces. Am. J. Respir. Crit. Care Med..

[138] Fricker M., Deane A., Hansbro P.M. (2014). Animal models of chronic obstructive pulmonary disease. Expert Opin. Drug Discov..

[139] Churg A., Cosio M., Wright J.L. (2008). Mechanisms of cigarette smoke-induced COPD: Insights from animal models. Am. J. Physiol. Lung Cell. Mol. Physiol..

[140] Wright J.L., Churg A. (2008). Animal models of COPD: Barriers, successes, and challenges. Pulm. Pharmacol. Ther..

[141] Wright J.L., Cosio M., Churg A. (2008). Animal models of chronic obstructive pulmonary disease. Am. J. Physiol. Lung Cell. Mol. Physiol..

[142] Ito J.T., Cervilha D.A.B., Lourenco J.D., Goncalves N.G., Volpini R.A., Caldini E.G., Landman G., Lin C.J., Velosa A.P.P., Teodoro W.P.R. (2019). Th17/Treg imbalance in COPD progression: A temporal analysis using a CS-induced model. PLoS ONE.

[143] Lourenco J.D., Ito J.T., Cervilha D.A.B., Sales D.S., Riani A., Suehiro C.L., Genaro I.S., Duran A., Puzer L., Martins M.A. (2018). The tick-derived rBmTI-A protease inhibitor attenuates the histological and functional changes induced by cigarette smoke exposure. Histol. Histopathol..

[144] Beckett E.L., Stevens R.L., Jarnicki A.G., Kim R.Y., Hanish I., Hansbro N.G., Deane A., Keely S., Horvat J.C., Yang M. (2013). A new short-term mouse model of chronic obstructive pulmonary disease identifies a role for mast cell tryptase in pathogenesis. J. Allergy Clin. Immunol..

[145] Lopes F.D., Toledo A.C., Olivo C.R., Prado C.M., Leick E.A., Medeiros M.C., Santos A.B., Garippo A., Martins M.A., Mauad T. (2013). A comparative study of extracellular matrix remodeling in two murine models of emphysema. Histol. Histopathol..

[146] Lourenco J.D., Neves L.P., Olivo C.R., Duran A., Almeida F.M., Arantes P.M., Prado C.M., Leick E.A., Tanaka A.S., Martins M.A. (2014). A treatment with a protease inhibitor recombinant from the cattle tick (Rhipicephalus Boophilus microplus) ameliorates emphysema in mice. PLoS ONE.

[147] Rodrigues R., Olivo C.R., Lourenco J.D., Riane A., Cervilha D.A.B., Ito J.T., Martins M.A., Lopes F. (2017). A murine model of elastase- and cigarette smoke-induced emphysema. J. Bras. Pneumol..

[148] Almeida-Reis R., Theodoro-Junior O.A., Oliveira B.T.M., Oliva L.V., Toledo-Arruda A.C., Bonturi C.R., Brito M.V., Lopes F., Prado C.M., Florencio A.C. (2017). Plant Proteinase Inhibitor BbCI Modulates Lung Inflammatory Responses and Mechanic and Remodeling Alterations Induced by Elastase in Mice. BioMed Res. Int..

[149] Theodoro-Junior O.A., Righetti R.F., Almeida-Reis R., Martins-Oliveira B.T., Oliva L.V., Prado C.M., Saraiva-Romanholo B.M., Leick E.A., Pinheiro N.M., Lobo Y.A. (2017). A Plant Proteinase Inhibitor from Enterolobium contortisiliquum Attenuates Pulmonary Mechanics, Inflammation and Remodeling Induced by Elastase in Mice. Int. J. Mol. Sci..

[150] Robertoni F.S., Olivo C.R., Lourenco J.D., Goncalves N.G., Velosa A.P., Lin C.J., Flo C.M., Saraiva-Romanholo B.M., Sasaki S.D., Martins M.A. (2015). Collagenase mRNA Overexpression and Decreased Extracellular Matrix Components Are Early Events in the Pathogenesis of Emphysema. PLoS ONE.

[151] Silver F.H., Horvath I., Foran D.J. (2002). Mechanical implications of the domain structure of fiber-forming collagens: Comparison of the molecular and fibrillar flexibilities of the alpha1-chains found in types I-III collagen. J. Theor. Biol..

[152] Ito S., Ingenito E.P., Brewer K.K., Black L.D., Parameswaran H., Lutchen K.R., Suki B. (2005). Mechanics, nonlinearity, and failure strength of lung tissue in a mouse model of emphysema: Possible role of collagen remodeling. J. Appl. Physiol..

[153] Wang Z., Li R., Zhong R. (2018). Extracellular matrix promotes proliferation, migration and adhesion of airway smooth muscle cells in a rat model of chronic obstructive pulmonary disease via upregulation of the PI3K/AKT signaling pathway. Mol. Med. Rep..

[154] Toledo A.C., Magalhaes R.M., Hizume D.C., Vieira R.P., Biselli P.J., Moriya H.T., Mauad T., Lopes F.D., Martins M.A. (2012). Aerobic exercise attenuates pulmonary injury induced by exposure to cigarette smoke. Eur. Respir. J..

[155] Gomes R.F., Shen X., Ramchandani R., Tepper R.S., Bates J.H. (2000). Comparative respiratory system mechanics in rodents. J. Appl. Physiol..

[156] Foronjy R.F., Mercer B.A., Maxfield M.W., Powell C.A., D’Armiento J., Okada Y. (2005). Structural emphysema does not correlate with lung compliance: Lessons from the mouse smoking model. Exp. Lung Res..

[157] Bellani G., Laffey J.G., Pham T., Fan E., Brochard L., Esteban A., Gattinoni L., van Haren F., Larsson A., McAuley D.F. (2016). Epidemiology, Patterns of Care, and Mortality for Patients With Acute Respiratory Distress Syndrome in Intensive Care Units in 50 Countries. JAMA.

[158] Amato M.B., Meade M.O., Slutsky A.S., Brochard L., Costa E.L., Schoenfeld D.A., Stewart T.E., Briel M., Talmor D., Mercat A. (2015). Driving pressure and survival in the acute respiratory distress syndrome. N. Engl. J. Med..

[159] Papazian L., Doddoli C., Chetaille B., Gernez Y., Thirion X., Roch A., Donati Y., Bonnety M., Zandotti C., Thomas P. (2007). A contributive result of open-lung biopsy improves survival in acute respiratory distress syndrome patients. Crit. Care Med..

[160] Pelosi P., Caironi P., Gattinoni L. (2001). Pulmonary and extrapulmonary forms of acute respiratory distress syndrome. Semin. Respir. Crit. Care Med..

[161] Hoelz C., Negri E.M., Lichtenfels A.J., Concecao G.M., Barbas C.S., Saldiva P.H., Capelozzi V.L. (2001). Morphometric differences in pulmonary lesions in primary and secondary ARDS. A preliminary study in autopsies. Pathol. Res. Pract..

[162] Petersen A.M., Pedersen B.K. (2005). The anti-inflammatory effect of exercise. J. Appl. Physiol..

[163] Meduri G.U., Kohler G., Headley S., Tolley E., Stentz F., Postlethwaite A. (1995). Inflammatory cytokines in the BAL of patients with ARDS. Persistent elevation over time predicts poor outcome. Chest.

[164] Zhou X., Dai Q., Huang X. (2012). Neutrophils in acute lung injury. Front. Biosci..

[165] Huang W., McCaig L.A., Veldhuizen R.A., Yao L.J., Lewis J.F. (2005). Mechanisms responsible for surfactant changes in sepsis-induced lung injury. Eur. Respir. J..

[166] Burnham E.L., Janssen W.J., Riches D.W., Moss M., Downey G.P. (2014). The fibroproliferative response in acute respiratory distress syndrome: Mechanisms and clinical significance. Eur. Respir. J..

[167] Chen Q., Luo A.A., Qiu H., Han B., Ko B.H., Slutsky A.S., Zhang H. (2014). Monocyte interaction accelerates HCl-induced lung epithelial remodeling. BMC Pulm. Med..

[168] Tomashefski J.F. (2000). Pulmonary pathology of acute respiratory distress syndrome. Clin. Chest Med..

[169] Forel J.M., Guervilly C., Farnarier C., Donati S.Y., Hraiech S., Persico N., Allardet-Servent J., Coiffard B., Gainnier M., Loundou A. (2018). Transforming Growth Factor-beta1 in predicting early lung fibroproliferation in patients with acute respiratory distress syndrome. PLoS ONE.

[170] Rubenfeld G.D., Herridge M.S. (2007). Epidemiology and outcomes of acute lung injury. Chest.

[171] Herridge M.S., Tansey C.M., Matte A., Tomlinson G., Diaz-Granados N., Cooper A., Guest C.B., Mazer C.D., Mehta S., Stewart T.E. (2011). Functional disability 5 years after acute respiratory distress syndrome. N. Engl. J. Med..

[172] Negri E.M., Montes G.S., Saldiva P.H., Capelozzi V.L. (2000). Architectural remodelling in acute and chronic interstitial lung disease: Fibrosis or fibroelastosis?. Histopathology.

[173] Gonzalez-Lopez A., Albaiceta G.M. (2012). Repair after acute lung injury: Molecular mechanisms and therapeutic opportunities. Crit. Care.

[174] Santos F.B., Nagato L.K., Boechem N.M., Negri E.M., Guimaraes A., Capelozzi V.L., Faffe D.S., Zin W.A., Rocco P.R. (2006). Time course of lung parenchyma remodeling in pulmonary and extrapulmonary acute lung injury. J. Appl. Physiol..

[175] Kamp R., Sun X., Garcia J.G. (2008). Making genomics functional: Deciphering the genetics of acute lung injury. Proc. Am. Thorac. Soc..

[176] Burnham E.L., Moss M., Ritzenthaler J.D., Roman J. (2007). Increased fibronectin expression in lung in the setting of chronic alcohol abuse. Alcohol. Clin. Exp. Res..

[177] Esper A., Burnham E.L., Moss M. (2006). The effect of alcohol abuse on ARDS and multiple organ dysfunction. Minerva Anestesiol..

[178] Aytacoglu B.N., Calikoglu M., Tamer L., Coskun B., Sucu N., Kose N., Aktas S., Dikmengil M. (2006). Alcohol-induced lung damage and increased oxidative stress. Respiration.

[179] Clark J.G., Milberg J.A., Steinberg K.P., Hudson L.D. (1995). Type III procollagen peptide in the adult respiratory distress syndrome. Association of increased peptide levels in bronchoalveolar lavage fluid with increased risk for death. Ann. Intern. Med..

[180] Farjanel J., Hartmann D.J., Guidet B., Luquel L., Offenstadt G. (1993). Four markers of collagen metabolism as possible indicators of disease in the adult respiratory distress syndrome. Am. Rev. Respir. Dis..

[181] Meduri G.U., Tolley E.A., Chinn A., Stentz F., Postlethwaite A. (1998). Procollagen types I and III aminoterminal propeptide levels during acute respiratory distress syndrome and in response to methylprednisolone treatment. Am. J. Respir. Crit. Care Med..

[182] Hamon A., Scemama U., Bourenne J., Daviet F., Coiffard B., Persico N., Adda M., Guervilly C., Hraiech S., Chaumoitre K. (2019). Chest CT scan and alveolar procollagen III to predict lung fibroproliferation in acute respiratory distress syndrome. Ann. Intensive Care.

[183] Thille A.W., Esteban A., Fernandez-Segoviano P., Rodriguez J.M., Aramburu J.A., Vargas-Errazuriz P., Martin-Pellicer A., Lorente J.A., Frutos-Vivar F. (2013). Chronology of histological lesions in acute respiratory distress syndrome with diffuse alveolar damage: A prospective cohort study of clinical autopsies. Lancet Respir. Med..

[184] Heyland D.K., Groll D., Caeser M. (2005). Survivors of acute respiratory distress syndrome: Relationship between pulmonary dysfunction and long-term health-related quality of life. Crit. Care Med..

[185] McHugh L.G., Milberg J.A., Whitcomb M.E., Schoene R.B., Maunder R.J., Hudson L.D. (1994). Recovery of function in survivors of the acute respiratory distress syndrome. Am. J. Respir. Crit. Care Med..

[186] Zheng S., Wang Q., D’Souza V., Bartis D., Dancer R., Parekh D., Gao F., Lian Q., Jin S., Thickett D.R. (2018). ResolvinD1 stimulates epithelial wound repair and inhibits TGF-beta-induced EMT whilst reducing fibroproliferation and collagen production. Lab. Investig..

[187] Bittencourt-Mernak M.I., Pinheiro N.M., Santana F.P., Guerreiro M.P., Saraiva-Romanholo B.M., Grecco S.S., Caperuto L.C., Felizardo R.J., Camara N.O., Tiberio I.F. (2017). Prophylactic and therapeutic treatment with the flavonone sakuranetin ameliorates LPS-induced acute lung injury. Am. J. Physiol. Lung Cell. Mol. Physiol..

[188] de Souza Xavier Costa N., Ribeiro Junior G., Dos Santos Alemany A.A., Belotti L., Zati D.H., Frota Cavalcante M., Matera Veras M., Ribeiro S., Kallas E.G., Nascimento Saldiva P.H. (2017). Early and late pulmonary effects of nebulized LPS in mice: An acute lung injury model. PLoS ONE.

[189] Oliveira V.R., Uriarte J.J., Falcones B., Zin W.A., Navajas D., Farre R., Almendros I. (2019). Escherichia coli lipopolysaccharide induces alveolar epithelial cell stiffening. J. Biomech..

[190] Park J.R., Lee H., Kim S.I., Yang S.R. (2016). The tri-peptide GHK-Cu complex ameliorates lipopolysaccharide-induced acute lung injury in mice. Oncotarget.

[191] Pinheiro N.M., Santana F.P., Almeida R.R., Guerreiro M., Martins M.A., Caperuto L.C., Camara N.O., Wensing L.A., Prado V.F., Tiberio I.F. (2017). Acute lung injury is reduced by the alpha7nAChR agonist PNU-282987 through changes in the macrophage profile. FASEB J..

[192] Simcock D.E., Kanabar V., Clarke G.W., Mahn K., Karner C., O’Connor B.J., Lee T.H., Hirst S.J. (2008). Induction of angiogenesis by airway smooth muscle from patients with asthma. Am. J. Respir. Crit. Care Med..

